# Exploring the potential link between ΔFosB and *N*-acetylcysteine in craving/relapse dynamics: can *N*-acetylcysteine stand out as a possible treatment candidate?

**DOI:** 10.1017/neu.2024.38

**Published:** 2024-10-17

**Authors:** Shokouh Arjmand, Mehran Ilaghi, Mohammad Shafie’ei, Pedro H. Gobira, Rodrigo Grassi-Oliveira, Gregers Wegener

**Affiliations:** 1 Translational Neuropsychiatry Unit, Department of Clinical Medicine, Aarhus University, Aarhus, Denmark; 2 Institute of Neuropharmacology, Kerman Neuroscience Research Center, Kerman University of Medical Sciences, Kerman, Iran; 3 Faculty of Medicine, Kerman University of Medical Sciences, Kerman, Iran; 4 Department of Affective Disorders, Aarhus University Hospital–Psychiatry, Aarhus, Denmark

**Keywords:** ΔFosB, N-acetylcysteine, relapse, substance use disorders, reinstatement

## Abstract

From a neuroscientific point of view, one of the unique archetypes of substance use disorders is its road to relapse, in which the reward system plays a crucial role. Studies on the neurobiology of substance use disorders have highlighted the central role of a protein belonging to the Fos family of transcription factors, ΔFosB. Relying on the roles ΔFosB plays in the pathophysiology of substance use disorders, we endeavour to present some evidence demonstrating that *N*-acetylcysteine, a low-cost and well-tolerated over-the-counter medicine, may influence the downstream pathway of ΔFosB, thereby serving as a treatment strategy to mitigate the risk of relapse in cases of substance use.

Summation

- ΔFosB is a critical component in relapse and reinstatement to substance use disorders that is highly expressed after repeated chronic administration of drugs of abuse, targetting glutamate release, spine density, transcriptional factors, and epigenetic mechanisms.
- Modulation of ΔFosB’s targets and upstream pathways might be a strategy to prevent relapse.
- Evidence suggests that N-acetylcysteine can potentially help reduce drug use relapse and craving. Here, we explored potential mechanisms through which N-acetylcysteine impacts dendritic arborisation, synaptic plasticity, transcriptional downstream targets, and epigenetics. These mechanisms may provide a possible link to ΔFosB-related signalling in drug use relapse and craving.

Perspective

- N-acetylcysteine influences glutamatergic and dopaminergic neurotransmission and modulates downstream signalling transcription factors and pathways altered by ΔFosB, making it a promising treatment option for preventing relapse in substance use.
- N-acetylcysteine’s safety profile, tolerability, and accessibility give this compound a significant advantage for use in this context, particularly since patients with substance use disorders are prone to the risk of overdose.
- Further extensive and robust preclinical and clinical research is needed to confirm the efficacy of N-acetylcysteine and to uncover the underpinning mechanisms behind its effects.

## A glance at the reward system in substance use disorders

Although different drugs of abuse act on distinct neurotransmitter systems of the brain and engender various psychoactive effects, all of them converge on the brain’s reward system (Nestler, 2005). Preclinical studies on substances abused by humans have provided us with better insights into addiction’s cellular and molecular pathways (Lynch *et al*., 2010). Studies on experimental animals have shown that both acute and chronic drug self-administration dramatically enhance the firing of the dopaminergic neurons of the ventral tegmental area (VTA) of the midbrain, an area with reciprocal projections to and from the mesocortical and mesolimbic pathways, including the nucleus accumbens (NAc) of the limbic forebrain, amygdala, cingulate gyrus, basal ganglia, and prefrontal cortex (PFC) (Nestler, 2005; Willuhn *et al*., 2010; Volkow and Morales, 2015).

All drugs of abuse are potentially rewarding. The NAc, amygdala (particularly the basolateral amygdala), ventromedial prefrontal cortex (including the orbitofrontal cortex), and posterior cingulate cortex are among the cortical and subcortical areas involved in the valuation and reward process. These areas evaluate the value, history, and cost of a reward and whether a rewarding activity needs to be repeated (Nestler, 2005; Wassum and Izquierdo, 2015; Loganathan and Ho, 2021). Increased VTA’s dopaminergic signalling will cause a sudden rise in the cyclic AMP (cAMP) and Ca^2+^ concentration in the NAc, following the activation of D_1_ receptors and, therefore, activate adenylyl cyclase (Muschamp and Carlezon, 2013). All these events subsequently lead to the activation of the cAMP response element-binding protein (CREB) through the phosphorylation of Ser^133^ (Muschamp and Carlezon, 2013).

CREB is a transcription factor that can either enhance or repress the expression of several genes. Phosphorylation of CREB at Ser^133^ results in the increased expression of dynorphin, which occupies kappa opioid receptors located on the VTA neurons and hampers the dopamine signalling of the mesocorticolimbic pathway of the reward circuitry. Thus, tolerance and dependence instigate, causing patients with substance use disorders to use more of a substance (Nestler, 2005; Muschamp and Carlezon, 2013).

On the other side, with chronic use of addictive substances, several structural modifications and many cellular adaptations will occur (Robinson and Kolb, 1997; Spiga *et al*., 2014; Zhang *et al*., 2017). Dopamine signalling of the VTA–NAc pathway also produces another protein named ΔFosB, which leads to the activation of several other genes switched on by phosphorylated CREB and suppression of dynorphin synthesis (McClung and Nestler, 2003; Nestler, 2005, 2012). Activation of these genes will result in the production of proteins in charge of responses sensitised to drugs of abuse, including nuclear factor kappa B (NF-κB) and cyclin-dependent kinase 5 (Cdk5), both of which can also result in structural alterations of the NAc, making it hypersensitive to addictive substances and drug-related cues (McClung and Nestler, 2003; Nestler, 2005, 2012). In this stage, the hippocampus also plays a part in the formation of context-specific memories of such experiences to remember the who, the where, the when, and the how of drug self-administration (contextual conditioning) and altogether build a road to relapse (Goodman and Packard, 2016; Silva *et al*., 2016). ΔFosB also contributes to the growth of NAc’s dendritic spines where it has been generated, and these sprouted branches of NAc’s dendritic spines make it more sensitized to the VTA signalling and will last even months after degradation of ΔFosB (Maze *et al*., 2010; Nestler, 2005, 2012). Upon withdrawal or abstinence, this established hypersensitivity and strong memory will result in craving, compulsive drug-seeking behaviours, and relapse (Nestler, 2005; Milton and Everitt, 2012).

**Figure 1.**
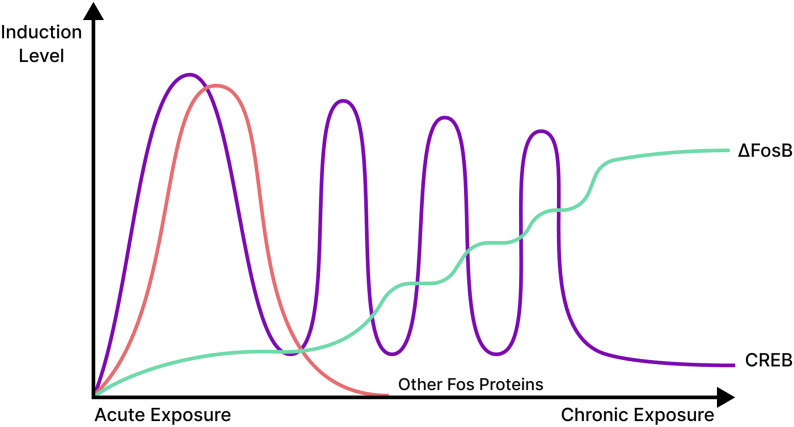
Temporal induction of ΔFosB, CREB, and other Fos proteins. Other Fos proteins (shown in orange) are rapidly induced by acute drug exposure and face a rapid decline afterward, while ΔFosB (shown in green) gradually increases and may persist for days. After a rapid increase following acute drug exposure, cAMP response element-binding protein (CREB; shown in purple) goes through a fluctuating pattern in chronic drug exposure until it wanes after days of abstinence.

After days of abstinence, the level of CREB remarkably wanes, while ΔFosB’s level, due to its considerable long half-life, will remain steady and reach a plateau for weeks and months even when substance use has ceased. Hence, the diminished concentration of CREB will open a door for the domination of ΔFosB’s deteriorating effects (Figure 1), leading to craving and drug-seeking behaviours (Nestler, 2005, 2012).

We are still in search of efficient, improved treatment strategies to markedly hamper craving and reduce relapse and reinstatement associated with chronic use of drugs of abuse. Having better insights into the driving forces behind reinstatement can potentially shed light on the better management of substance use disorders in the future. Based on the accumulating evidence on ΔFosB’s role in substance use relapse, the following section digs deep into how ΔFosB plays a crucial role in substance use relapse, laying the foundation for understanding how targeting this molecule and its associated signalling pathways could be a way to lessen craving.

## ΔFosB as a molecular target in substance use disorders treatment

ΔFosB is a 33–37 kDa protein that belongs to the Fos family of transcription factors, which can form either a heterodimer with the Jun family of proteins, particularly JunD or form stable molecular assemblies on its own (Wang *et al*., 2012; Yin *et al*., 2020). Such heterodimerization shapes functional activator protein 1 (AP-1) complexes that can be attached to the AP-1 sites, regulating the transcription of various genes (Nestler, 2001, 2008).

**Figure 2.**
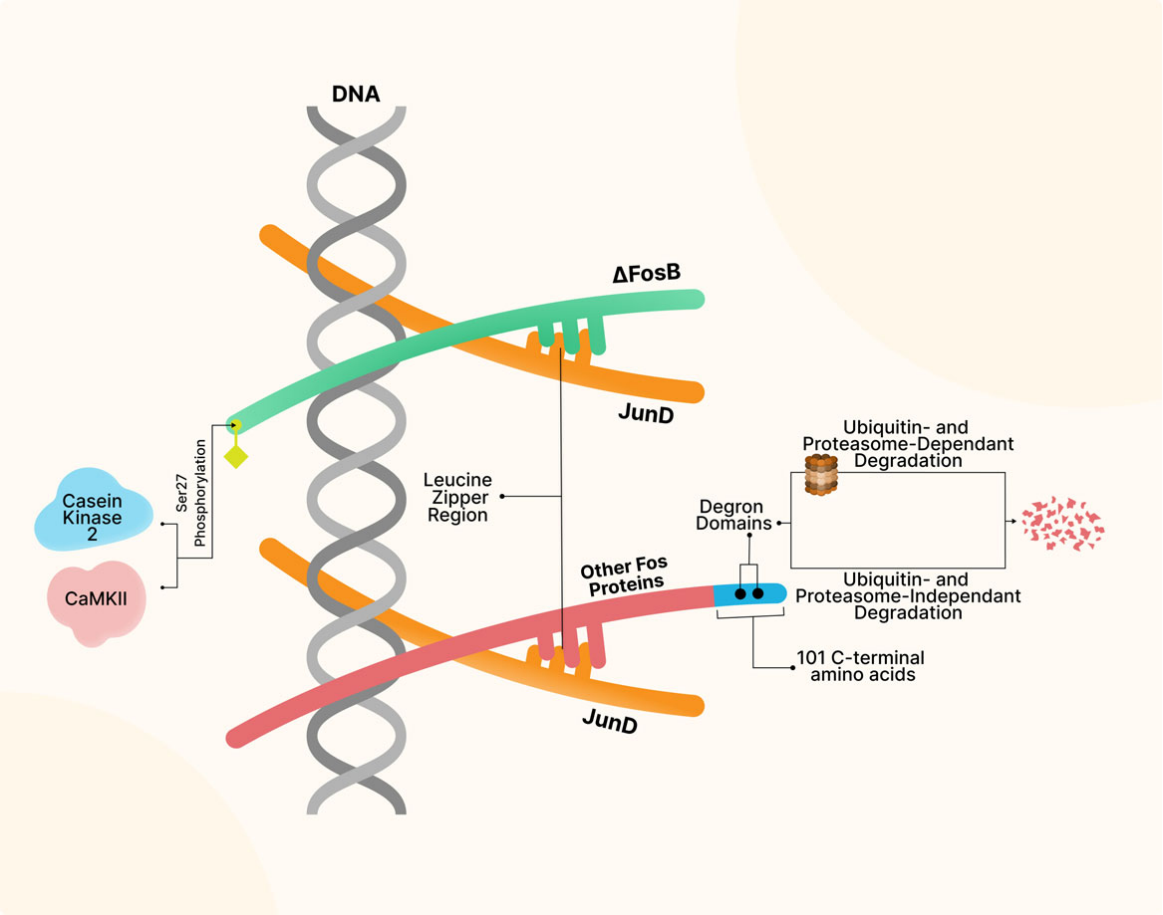
Structure of ΔFosB and other Fos proteins. Compared to other Fos proteins, ΔFosB lacks the 101 C-terminal amino acids and the degron domains responsible for ubiquitin- and proteasome-dependent and independent degradation, thus enhancing its stability. Additionally, Ser27 phosphorylation by Casein Kinase 2 and Ca^2+^/calmodulin-dependent protein kinase (CaMKII) prevents decomposition of this protein, leading to its long-term half-life.

Distinct from other FosB proteins, ΔFosB is bereft of 101 C-terminal amino acids, making it lack two degron domains and, consequently, less prone to degradation (Figure 2) (Carle *et al*., 2007; Wang *et al*., 2012; Zhang *et al*., 2014). Moreover, phosphorylation of a well-preserved serine residue (Ser27) of ΔFosB *via* either previously surmised casein kinase 2 (Ulery *et al*., 2006) or more recently explored Ca^2+^/calmodulin-dependent protein kinase (CaMKII) (Robison *et al*., 2013) prevents degradation of this protein, leading to its long half-life (around 8 days in vivo) (Figure 2). Such longevity confers disparate responses in gene expression after acute and chronic administration of drugs of abuse and makes ΔFosB stable enough to remain active for several weeks even after cessation of drug exposure or drug withdrawal (Figure 1) (Nestler, 2008). Data have shown that such durability is not correlated with ΔFosB’s mRNA long life but rather the protein per se (Kelz and Nestler, 2000).

Unlike most Fos family members whose expression is triggered by acute exposure to drugs of abuse and only lasts for hours, ΔFosB is highly expressed in the striatal regions in a medium spiny neuron (MSN)-subtype-selective pattern after repeated chronic administration (Olsen, 2011; Lobo *et al*., 2013a; Zhang *et al*., 2014). Accordingly, numerous drugs of abuse induce ΔFosB only in MSNs-expressing dopamine D1 receptors, whereas others, such as opioids, induce ΔFosB equally in D1- and D2-MSNs (Lobo *et al*., 2013b). Similarly, a recent study has shown that chronic cocaine use induced broad changes in ΔFosB binding in both D1- and D2-MSNs in NAc (Yeh *et al*., 2023). On the other hand, prolonged administration of some antipsychotic medications induces ΔFosB exclusively in D2-MSNs (Lobo *et al*., 2013b).

Additionally, recent data indicate that, besides the MSN subtype, ΔFosB mediates distinct transcriptional effects in males and females (Lardner *et al*., 2021). Induction of ΔFosB in each subtype of MSNs (D1- vs. D2-MSNs) led to sex- and MSN-specific control of transcripts. In female NAc, induction of ΔFosB in D1-MSNs resulted in opposite regulation compared to D2-MSNs (Lardner *et al*., 2021). Moreover, little overlap was seen among regulatory downstream genes when males and females were compared after induction of ΔFosB in each specific MSN subtype (Lardner *et al*., 2021). Although the sex-specific transcriptional effects of ΔFosB are less studied, these findings are of utmost importance since there are notable sex differences throughout the progression of various substance use disorders and their comorbid conditions (Daiwile *et al*., 2022b; Chapp *et al*., 2022a, 2024). Although women tend to be engaged with drugs less frequently than men, they often progress more rapidly to meet the criteria for substance use disorders and may experience more severe psychiatric disturbances compared to men (Cadet, 2021). Additionally, women are generally more prone to relapse (for a comprehensive review, see (Becker and Koob, 2016)). Therefore, the preliminary findings of Lardner et al., on sex-specific transcriptional effects of ΔFosB have significant implications for understanding the pathophysiology underlying relapse and the use of potential drugs in preventing relapse (Lardner *et al*., 2021).

Although it has been shown that ΔFosB’s distribution in the different parts of the striatum is diverse when various drugs are abused, nearly all of them induce the accumulation of ΔFosB in the NAc (Perrotti *et al*., 2008). Besides, findings have suggested that the accumulation of ΔFosB induced by addictive substances targets the dynorphin-containing class of MSNs (Kelz and Nestler, 2000).

Gajewski et al., have also demonstrated that the expression profile of ΔFosB differs in various brain regions associated with substance use disorders. They reported a reduced level of ΔFosB in the hippocampus but not in the prefrontal cortex, which is supported by the observation that even some investigated upstream target genes, such as GluA2 and CaMKII, are also downregulated solely in the hippocampus (Gajewski *et al*., 2016).

These observations suggest that ΔFosB could be one of the main contributing molecules of reinstatement. In this regard, studies on animal models of relapse have demonstrated that overexpression of ΔFosB not only sensitises the rewarding effect of drugs of abuse but also produces increased drug-seeking behaviour that leads to relapse (Kelz *et al*., 1999; Colby *et al*., 2003; Zachariou *et al*., 2006). Of note, McClung et al., have implied that short or long-term expression of ΔFosB acts oppositely and thus leaves distinct outcomes (McClung and Nestler, 2003). They showed that ΔFosB’s short-term expression in the NAc upregulates many of the same genes as CREB, presumably *via* direct effect. However, the scenario turned back in case of either overexpression or prolonged elevation of ΔFosB, which facilitates the induction of transcriptional downregulation of genes that CREB had upregulated and vice versa (McClung and Nestler, 2003).

Besides preclinical studies, post-mortem analyses of the brain tissues of chronic opioid abusers have revealed enhanced expression of ΔFosB in the NAc that was followed by a rise in the level of its downstream targets such as Cdk5, NF-κB, CREB, brain-derived neurotrophic factor, and JunD in both the hippocampus and NAc (Seltenhammer *et al*., 2016). They confirmed that ΔFosB and its downstream transcriptional targets are critical to inducing sustainable brain changes and establishing a strong memory associated with drugs of abuse on the road to dependency and relapse (Seltenhammer *et al*., 2016).

The dimerisation of ΔFosB with JunD possibly circumvents CREB on being attached to the cAMP response element (CRE) site and, as a result, acts contrariwise to that of CREB. It can also be postulated that higher levels of ΔFosB may act as an AP-1 activator (McClung and Nestler, 2003). Finally, CaMKII, a protein upregulated as ΔFosB is continuously overexpressed, has been introduced as another potential candidate for these observations (McClung and Nestler, 2003; Robison *et al*., 2013). CaMKII hinders the dimerisation of CREB with CREB binding protein via phosphorylating CREB (McClung and Nestler, 2003).

In a study conducted by Vialou et al., following chronic cocaine administration, expression of both CREB and serum response factor (SRF) were shown to be essential for the accumulation and regulation of NAc’s ΔFosB (Vialou *et al*., 2012; Eagle *et al*., 2019). They concluded that upstream modulation of ΔFosB requires the adequate expression of both transcription factors and deletion of either could not prevent the accumulation of ΔFosB in the NAc (Vialou *et al*., 2012; Eagle *et al*., 2019).

There are also studies pointing to RNA-binding proteins involved in regulating ΔFosB. Recent evidence suggests synergistic effects of D1 dopaminergic and activin receptor-like kinase 4 (ALK4) signalling, mediated by activation of poly-binding protein 1 (PCBP1) and Smad3 in MSNs of NAc, induce ΔFosB production and its reward-related behaviour (Krapacher *et al*., 2022). Enhanced expression of an RNA-binding protein called polypyrimidine tract-binding protein 1 (PTBP1) was suggested to cause reduced transcription of ΔFosB (Alibhai *et al*., 2007; Bryant and Yazdani, 2016). Another study pointed out that elevation in the level of ΔFosB can be modulated by intraperitoneal administration of molecular hydrogen in methamphetamine-dependent mice (Wen *et al*., 2020). Wen et al. also showed that molecular hydrogen (delivered by the administration of hydrogen-rich saline) reduces behavioural sensitisation by acting as an antioxidant, inhibiting the production of superoxide anion, and diminishing the amount of phosphorylated ERK and ΔFosB in the NAc (Wen *et al*., 2020).

Interestingly, this observed overexpression is not only limited to the drugs of abuse but also other compounds, such as sweeteners that activate the brain’s reward system (Salaya-Velazquez *et al*., 2020) and even stressful conditions (Perrotti *et al*., 2004; Scalize Hirata *et al*., 2019). Keeping all these in mind, it is worth mentioning that stress and stressful adverse events in life can not only lead to addiction but also predispose abstained patients with substance use disorders to reinstate and relapse (Goeders, 2003; Sinha, 2008, 2012). Though different mechanisms contribute to such phenomena, assessing the role stress plays in manipulating ΔFosB toward an increased susceptibility to relapse is not out of reason. A study has shown that repeated social defeat stress significantly enhances the expression of ΔFosB in the frontal cortex, shell, and core of the NAc, and the medial, central, and basolateral amygdala (mesocorticolimbic areas) that started after the last imposed stress and lasted for 21 days which was correlated with upregulation of µ-opioid receptor in the VTA-NAc pathway (Nikulina *et al*., 2008). A report also indicates that chronic restraint stress induces higher locomotor activity after amphetamine administration, resulting in cross-sensitization that is associated with enhanced ΔFosB expression levels in the NAc of only adult and not adolescent rats (Carneiro De Oliveira *et al*., 2016).

## A path to relapse: a concise look into possible mechanisms in which ΔFosB begets drug-seeking behaviours and craving and how N-acetylcysteine may rectify these alterations

**Figure 3.**
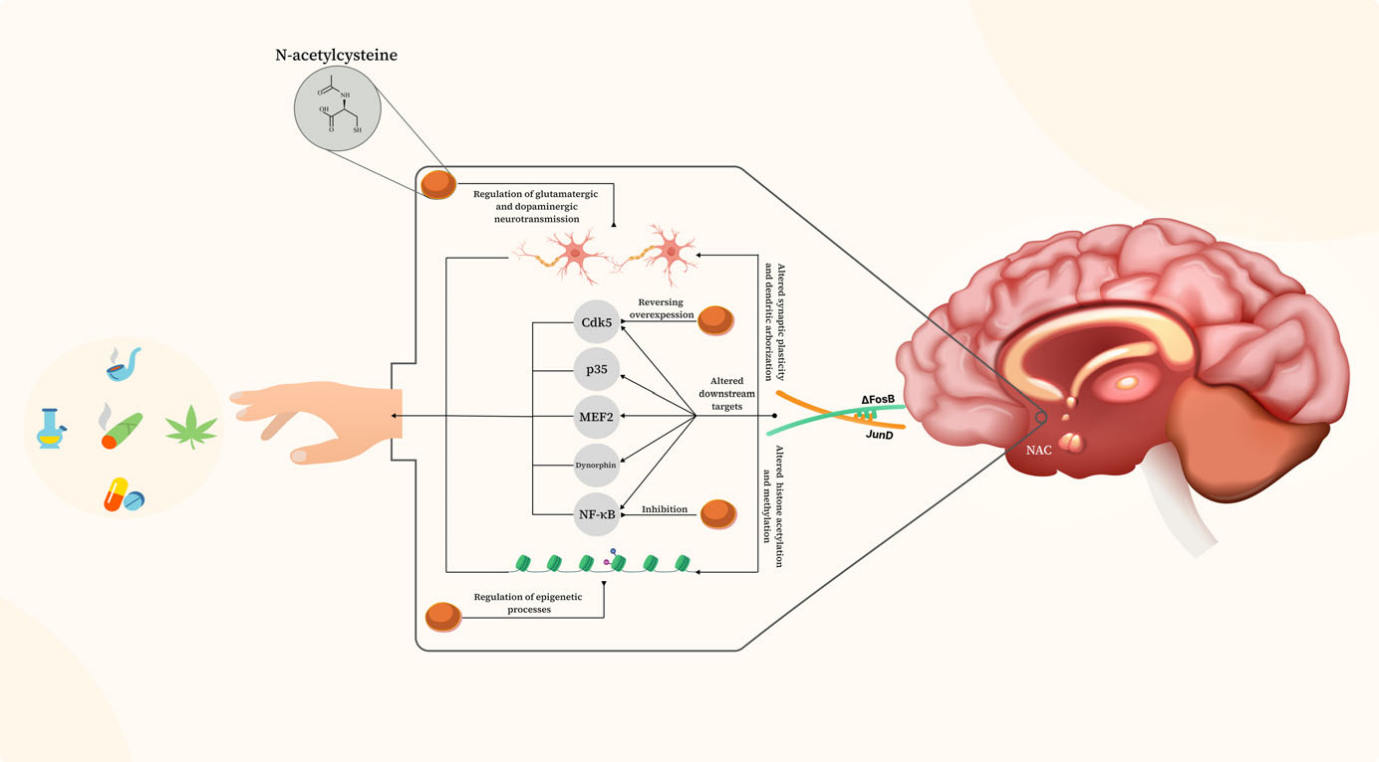
Mechanisms of ΔFosB involvement in relapse and rectifying actions of N-acetylcysteine. ΔFosB contributes to drug-seeking behaviours and relapse through various mechanisms, including enhanced expression of glutamatergic receptors, increasing spine density of medium spiny neurons, modulation of downstream transcription factors [including cyclin-dependent kinase 5 (Cdk5), p35, dynorphin, nuclear factor kappa B (NF-kB), and presumably myocyte enhancer factor 2 (MEF2)] and through epigenetic mechanisms namely by altering histone acetylation and methylation. N-acetylcysteine influences these signaling pathways, holding the potential to reduce drug craving and mitigate the risk of relapse.

Human studies are indicative that behavioural changes associated with addiction are exceptionally persistent. Therefore, researchers started figuring out the cellular and molecular mechanisms involved in enduring behavioural abnormalities. In this section, we briefly enumerate potential targets that ΔFosB aims at on various levels and determine whether N-acetylcysteine could alter these pathways (Figure 3).

N-acetylcysteine is an accessible, well-tolerated, over-the-counter medicine of low cost with a safe profile regarding adverse effects. It serves as the N-acetylated derivative of the amino acid cysteine and, as such, acts as a prodrug by binding to the cysteine-glutamate exchanger. N-acetylcysteine also promotes the efflux of glial glutamate while importing cysteine, resulting in a net increase in the extracellular levels of glutamate in the brain.

### Dendritic arborisation and synaptic plasticity as common targets of different pathways

ΔFosB is presumed to influence AMPAR-containing GluR2 receptors and modify glutamatergic signalling by altering the neural excitability of NAc neurons due to overexpression of these receptors. Reduced neural conductance followed by diminished Ca^2+^ permeability can affect the excitability of neurons, and prolonged exposure to addictive drugs can change the susceptibility of neurons to rewarding stimuli (Kelz *et al*., 1999; Nestler, 2001; Nestler *et al*., 2001). This phenomenon has been shown to exacerbate responses to cues and subsequent increases in craving and relapse (Kelz *et al*., 1999; Todtenkopf *et al*., 2006). An additional study exploring the mechanisms underlying resilience and antidepressant response has further affirmed that ΔFosB can regulate the expression of GluR2 and exert control over complex behaviours (Vialou *et al*., 2010). However, in a preclinical study conducted by Conrad et al., a significant connection was established between the presence of increased GluR2-lacking AMPARs in the NAc of rats following extended periods of withdrawal from cocaine, leading to the hypothesis that such an increase may also contribute to enhanced cocaine craving (Conrad *et al*., 2008).

Short-term expression of ΔFosB also leaves different outcomes on D1-type versus D2-type MSNs of the NAc (Grueter *et al*., 2013). Observations indicate diminished AMPA-related synaptic transmission (reduced excitatory synaptic strength) in D1 receptor-expressing neurons in both the NAc’s shell and core via the direct pathway that shapes silent synapses (increased silent synapses onto D1-type). In contrast, an increase in D2-type excitatory transmission through an indirect pathway only on the shell of NAc has been reported (Grueter *et al*., 2013). Grueter et al. further depicted that morphological changes of the immature dendritic spines are under the influence of direct D1-type MSNs of the NAc and attributed the cocaine-evoked abnormal behaviours to the D1 direct but not D2 indirect pathway, therefore highlighting the cell-type and sub-regional-specific manner of ΔFosB’s mode of action to cocaine-associated behavioural responses (Grueter *et al*., 2013). In contrast, Lee et al., have demonstrated that ΔFosB participates in the short-term induction of increased spine density in both D1- and D2-containing MSNs of NAc (Lee *et al*., 2006). However, such an enhanced density of dendritic spines and ΔFosB overexpression are maintained and remain stable, specifically in the D1 type MSNs of NAc, even after a month of cocaine withdrawal (Lee *et al*., 2006). Moreover, elevated levels of ΔFosB can enhance the expression of NMDA receptors of the mesocorticolimbic pathway, especially on the MSNs of the NAc, which further leads to long-term potentiation and hijacks the associated memory pathways (Daneff and Jadavji, 2019).

### Transcriptional downstream targets

Evidence has pointed out that ΔFosB is one of the primary regulators of transcriptional phenotype changes that occur with repeated consumption of addictive substances that ultimately result in not only addiction-related behavioural responses but also natural rewards (Nestler, 2008; Wallace *et al*., 2008; Bali and Kenny, 2019).

Studies on transgenic animals overexpressing ΔFosB suggested the prominent role of this transcription factor in liability to relapse after the termination of drug consumption and even when the level of ΔFosB backs to its normal concentration (Colby *et al*., 2003; McClung and Nestler, 2003; Nestler, 2008). Findings on the expression profile of ΔFosB have delineated that the ongoing predisposition to relapse and drug-taking/seeking behaviours might not directly be affected by ΔFosB *per se* (Larson *et al*., 2010) but instead can trigger several transcriptional cascades that have impacts on the morphology and neural circuits of NAc neurons, thus mediating neural adaptation (Lee *et al*., 2006; Ang *et al*., 2008; Maze *et al*., 2010; Gajewski *et al*., 2016).

Enhanced expression and accumulation of ΔFosB raise levels of both mRNA and protein of Cdk5 and p35, which is regulated by the AP-1 site on the gene’s promoter (Chen *et al*., 2000; Bibb *et al*., 2001; Benavides and Bibb, 2004). Cdk5/p35 involves diverse central nervous system processes, including synaptic plasticity, learning, and memory (Takahashi *et al*., 2022). Cdk5 further dampens D1 dopaminergic signalling, possibly via augmented phosphorylation of dopamine- and cAMP-regulated neuronal phosphoprotein DARPP-32 (PPP1RB) (Bibb *et al*., 2001; Benavides and Bibb, 2004).

In addition, myocyte enhancer factor 2 (MEF2) has been introduced as another critical regulator of structural and behavioural plasticity and is involved in developing various neuropsychiatric disorders (Pulipparacharuvil *et al*., 2008; Zhang and Zhao, 2022). Reduction in the striatal level of MEF2 has been implicated in causing increased dendritic spine density after chronic administration of cocaine. This event is induced by the release of cAMP through the activation of D1 receptors that prevents the further activity of calcineurin at the inhibitory Cdk5 site (Pulipparacharuvil *et al*., 2008). However, the possible correlation between diminished MEF2 and increased expression of ΔFosB should be sought.

Additionally, the opioid peptide dynorphin is a target through which ΔFosB exerts its behavioural phenotype (Zachariou *et al*., 2006). It is also shown that prodynorphin, the dynorphin precursor, is a known target for CREB and that chronic consumption of cocaine is associated with CREB-mediated dynorphin expression in NAc (Teague and Nestler, 2021).

Another putative target of ΔFosB is another transcription factor named NF-κB. Enhanced expression of NF-κB in the NAc only after chronic but not acute treatment with cocaine has raised the idea that NF-κB may be under the control of ΔFosB. NF-κB engages in long-term plasticity and enhances dendritic density as Cdk5 does (Bibb *et al*., 2001; Ang *et al*., 2008; Boersma *et al*., 2011; Dresselhaus *et al*., 2018), thus contributing to craving and drug-seeking behaviours.

### Epigenetics

There are emerging lines of evidence that ΔFosB might leave its long-lasting effect by epigenetic alterations since studies have signified that even after protracted withdrawal and return of ΔFosB’s level to normal, the corresponding effects of it still abide (Mews *et al*., 2018).

ΔFosB alters neuronal morphology by targeting histone methyltransferase G9a. Maze et al. have reported that enhanced expression of ΔFosB can result in the repression of lysine dimethyltransferase G9a, which further reduces the dimethylation of histone 3 lysine 9 (H3K9) in the NAc during repeated cocaine exposure. Such a downregulated level of G9a and the subsequent decrease in the process of histone methylation leads to dendritic arborisation and an enhanced tendency to reinstate cocaine abuse (Maze *et al*., 2010).

Acetylation of H3 and H4 histone subtypes at the promoters of plasticity-associated genes, including Fos, has also been shown to be increased following cocaine exposure (Stewart *et al*., 2021). Furthermore, a recent study demonstrated that inhibition of histone deacetylase results in an increase of ΔFosB gene expression in the NAc and medial prefrontal cortex while also reducing reinstatement of morphine-induced conditioned place preference in rats, confirming the role of epigenetic mechanisms, namely histone acetylation, in the adjustment of drug-induced plasticity (Saberian *et al*., 2021). Promoters of ΔFosB and CREB genes involved in glutamate transmission are thought to be hyperacetylated in the NAc after cocaine administration (Kennedy *et al*., 2013), and sustained inhibition of histone deacetylase can stimulate G9a (Kennedy *et al*., 2013; Eagle *et al*., 2019). ΔFosB and CREB can facilitate signalling transmission through enhanced permeability of Ca^2+^ that is modulated by histone acetylation (Mews *et al*., 2018). By using H3.3 histone barcoding of NAc, Wimmer et al. reported an increase in upregulation of the FosB gene in cocaine self-administration mice and the implication of other novel molecular cascades in cocaine-induced neuronal plasticity (Wimmer *et al*., 2019). Moreover, following administration of amphetamine, histone deacetylase budges to the Fos gene promoter and controls its expression (Torres *et al*., 2015). Therefore, it should be explored in-depth whether ΔFosB can broadly affect histone modification and coding (Eagle *et al*., 2019).

## N-acetylcysteine and preclinical and clinical studies on relapse to substance abuse

There are comprehensive reviews on preclinical and clinical studies of N-acetylcysteine in substance use disorders and other psychiatric disorders (Dean *et al*., 2011; Minarini *et al*., 2017; Smaga *et al*., 2021a). Here, we only focus on evidence with a direct nexus to relapse/cravings and drug-seeking behaviours.

### Preclinical studies

The majority of studies investigating the effect of N-acetylcysteine on relapse or cravings have primarily focused on alcohol and cocaine use. In a preclinical study, N-acetylcysteine was able to attenuate the accumulation of ΔFosB in the mPFC, leading to reduced craving and prevention of ethanol-induced neuroadaptions associated with ΔFosB in a mouse model of behavioural sensitisation (Morais-Silva *et al*., 2016). Although, unlike most studies, the authors could not observe an increased ΔFosB expression in the NAc (Morais-Silva *et al*., 2016). Another preclinical report of such an effect was published later, indicating that N-acetylcysteine diminishes ethanol-seeking behaviour to a great extent of 77 percent along with reacquisition after protracted abstinence in rats self-administering ethanol chronically by 78 percent, pointing to the application of N-acetylcysteine as a treatment to prevent relapse (Lebourgeois *et al*., 2018).

Recently, Fredriksson et al., have also presented evidence that N-acetylcysteine improves impulse control and attenuates the likelihood of relapse in male rats with long-term alcohol consumption (Fredriksson *et al*., 2023). However, no significant difference was observed in the quantity of alcohol consumed or the motivation to drink (Fredriksson *et al*., 2023). Using another animal model to evaluate ethanol-relapse drinking behaviour, the alcohol deprivation effect model, N-acetylcysteine was shown to be effective in preventing relapse by reducing the heightened intake of alcohol following a period of abstinence (Cano-Cebrián *et al*., 2021). The effective dose range of N-acetylcysteine on ethanol in these preclinical studies was between 60 and 120 mg/kg.

Besides alcohol, some preclinical studies have also addressed N-acetylcysteine’s impact on cocaine-seeking behaviour. Findings from a study by Amen et al., demonstrated that repeated N-acetylcysteine administration (60 mg/kg for 7 days) resulted in a significant decrease in cocaine-seeking behaviour in rats, and these findings were further validated in the same study through reductions in craving in cocaine-dependent humans as well (Amen *et al*., 2011). Additionally, findings from another study have demonstrated that N-acetylcysteine (100 mg/kg) prevented cocaine and alcohol seeking following acute restraint stress (Garcia-Keller *et al*., 2020). In another study, N-acetylcysteine amide has also been shown to effectively block cocaine-seeking behaviour in rats (Jastrzębska *et al*., 2016). Moreover, N-acetylcysteine has been demonstrated to prevent cocaine-primed reinstatement, suggesting that this treatment can alter the plasticity-dependent effects of cocaine (Madayag *et al*., 2007).

### Clinical trials

Several clinical studies have also investigated the impact of N-acetylcysteine on craving and substance use relapse in populations with various substance use disorders (Table 1). In a human study involving veterans with both post-traumatic stress disorder (PTSD) and substance use disorders, the administration of N-acetylcysteine (1200 mg twice a day) led to improved craving compared to the placebo (Back *et al*., 2016). A recent double-blind placebo-controlled trial has also demonstrated that supplementation with N-acetylcysteine (2700 mg daily) resulted in significant benefits on measures of drug craving among treatment-resistant PTSD individuals. However, it did not affect other PTSD symptoms (Kanaan *et al*., 2023).

**Table 1.** Clinical trials exploring the effects of N-acetylcysteine on substance use abstinence, relapse, and craving

Studied population diagnosis	Trial design	N-acetylcysteine dose and duration	Findings	Reference
PTSD and substance use disorder	Double-blind randomised placebo-controlled trial	2400 mg/day for 8 weeks	N-acetylcysteine improved craving compared to the placebo	(Back *et al*., 2016)
PTSD	Double-blind randomised placebo-controlled trial	2700 mg/day for 12 weeks	N-acetylcysteine improved substance craving duration and resistance, but not other PTSD symptoms compared to the placebo	(Kanaan *et al*., 2023)
Cocaine use disorder	Double-blind randomised placebo-controlled trial	1200 or 2400 mg/day for 8 weeks	Individuals who were already abstinent before the trial’s commencement exhibited a longer duration of time to relapse and lower craving rates in the 2400 mg/day N-acetylcysteine group compared to the placebo	(LaRowe *et al*., 2013)
Cocaine use disorder	Double-blind placebo-controlled crossover trial	3600 mg/day for 1 week	N-acetylcysteine suppressed cocaine-seeking behaviour	(Woodcock *et al*., 2021)
Cocaine use disorder	Double-blind randomised placebo-controlled trial	2400 mg/day for 25 days	N-acetylcysteine did not affect craving, number of abstinent days and days until relapse. However, N-acetylcysteine was associated with lower positive urine tests, lower cocaine use-related problems and inhibition	(Schulte *et al*., 2018)
Cocaine use disorder	Double-blind placebo-controlled crossover trial	2400 mg/day for 3 days	There were trends for a greater reduction in withdrawal symptoms and craving within N-acetylcysteine treatment	(LaRowe *et al*., 2006)
Methamphetamine use disorder	Double-blind randomised placebo-controlled trial	2400 mg/day for 12 weeks	No reduction in craving, severity of dependence and withdrawal was seen in the N-acetylcysteine group compared to the placebo	(McKetin *et al*., 2021)
Methamphetamine use disorder	Double-blind placebo-controlled crossover trial	600 mg/day for 2 weeks followed by 1200 mg/day for 2 weeks	N-acetylcysteine resulted in a decrease in methamphetamine craving	(Mousavi *et al*., 2015)
Cannabis use disorder	Double-blind randomised placebo-controlled trial	2400 mg/day for 8 weeks	Adolescents receiving N-acetylcysteine had improved cannabis abstinence compared to placebo	(Gray *et al*., 2012)
Cannabis use disorder	Double-blind randomised placebo-controlled trial	1200 mg/day for 12 weeks	N-acetylcysteine and placebo groups did not differ in cannabis abstinence among adults	(Gray *et al*., 2017)
Nicotine use disorder	Double-blind randomised placebo-controlled trial	3600 mg/day for 3.5 days	Patients receiving N-acetylcysteine reported their initial smoking experience being less rewarding after abstinence, but no significant effects were seen in craving and withdrawal symptoms	(Schmaal *et al*., 2011)
Nicotine use disorder	Double-blind randomised placebo-controlled trial	2400 mg/day for 3.5 days	Smokers in N-acetylcysteine group reported less craving compared to placebo	(Froeliger *et al*., 2015)
Nicotine use disorder and pathological gambling	Double-blind randomised placebo-controlled trial	1200–3000 mg/day for 12 weeks	Significant benefit of N-acetylcysteine was seen in nicotine dependence scores compared to placebo	(Grant *et al*., 2014)
Nicotine use disorder	Double-blind randomised placebo-controlled trial	2400 mg/day for 4 weeks	Participants treated with N-acetylcysteine reported a reduction in cigarettes smoked	(Knackstedt *et al*., 2009)
Nicotine use disorder	Double-blind randomised placebo-controlled trial	3000 mg/day for 12 weeks	N-acetylcysteine significantly reduced the daily number of cigarettes used and resulted in greater quit rate	(Prado *et al*., 2015)
Nicotine use disorder	Double-blind randomised placebo-controlled trial	2400 mg/day for 2 weeks	N-acetylcysteine did not affect craving or withdrawal symptoms	(Schulte *et al*., 2017)
Nicotine use disorder	Double-blind randomised placebo-controlled trial	2400 mg/day for 4 weeks	N-acetylcysteine group had a higher abstinence rate and lower craving than the placebo group	(Harlivasari *et al*., 2024)
Nicotine use disorder	Double-blind randomised placebo-controlled trial	1800 mg/day for 16 weeks	There was no significant difference in smoking outcomes between N-acetylcysteine and placebo groups	(Arancini *et al*., 2021)
Nicotine use disorder	Double-blind randomised placebo-controlled trial	2400 mg/day for 8 weeks	No differences between N-acetylcysteine and placebo groups in terms of early abstinence, time to relapse and end-of-treatment abstinence	(McClure *et al*., 2021)
Alcohol use disorder	Double-blind randomised placebo-controlled trial	2400 mg/day for 4 weeks	N-acetylcysteine had effects on number of standard drinks per drinking day, but not on craving compared to placebo	(Morley *et al*., 2023)
Alcohol use disorder	Double-blind randomised placebo-controlled trial	1200 mg/day or 2400 mg/day for 5 days	N-acetylcysteine did not influence alcohol self-administration or other subjective effects	(Stoops *et al*., 2020)
Alcohol use in cannabis-dependent individuals	Double-blind randomised placebo-controlled trial	2400 mg/day for 12 weeks	N-acetylcysteine increased the odds of alcohol abstinence, fewer drinks per week and fewer drinking days per week	(Squeglia *et al*., 2018)
Alcohol use in cannabis-dependent individuals	Randomised placebo-controlled trial	2400 mg/day for 8 weeks	N-acetylcysteine did not affect abstinence and binge drinking days compared to the placebo. However, in the N-acetylcysteine group, but not the placebo group, less marijuana use was associated with less alcohol use	(Squeglia *et al*., 2016)

There have also been several studies exploring N-acetylcysteine in cocaine use disorder. In a double-blind placebo-controlled trial aiming at evaluating the efficacy of N-acetylcysteine (2400 mg daily) for treating cocaine use disorder, no correlations were found between measures related to relapse (LaRowe *et al*., 2013). Nevertheless, a subgroup of enrolled patients who were already abstinent before the trial’s commencement exhibited a longer duration of time to relapse, indicating that N-acetylcysteine might have a protective role in abstained patients with cocaine use disorder (LaRowe *et al*., 2013). Findings from the same authors also showed that there were trends for a greater reduction in cocaine withdrawal symptoms and craving within N-acetylcysteine treatment compared to the placebo (LaRowe *et al*., 2006). In a more recent trial by Woodcock et al., N-acetylcysteine treatment (3600 mg/day for 1 week) suppressed cocaine-seeking behaviour (Woodcock *et al*., 2021). However, another trial failed to show N-acetylcysteine’s effect on craving, number of abstinent days, and days until relapse in cocaine-dependent individuals. Nevertheless, the study showed that N-acetylcysteine was associated with lower positive urine tests, lower cocaine use-related problems and inhibition (Schulte *et al*., 2018).

Studies on the effects of N-acetylcysteine on methamphetamine use disorder have been inconclusive so far, with one study showing no reduction in craving (McKetin *et al*., 2021), but another study reporting a decrease in methamphetamine craving (Mousavi *et al*., 2015). Similarly, trials on cannabis use disorders have been inconsistent, with studies showing improved cannabis abstinence following N-acetylcysteine treatment in adolescents (gray *et al*., 2012), but not in adults (gray *et al*., 2017).

In terms of nicotine use disorder, in a pilot randomised placebo-controlled trial, no significant decrease in craving after short-term abstinence (3.5 days) was observed. However, participants receiving a daily dose of 3600 mg N-acetylcysteine reported their initial smoking experience being less rewarding after abstinence. This finding suggests a potential avenue for further research into the viability of N-acetylcysteine as a good candidate for relapse prevention in nicotine use disorder as well (Schmaal *et al*., 2011). In later studies, while some trials reported no significant outcomes following N-acetylcysteine in nicotine-dependent individuals (Schulte *et al*., 2017; McClure *et al*., 2021), several other trials reported that N-acetylcysteine treatment resulted in less craving (Froeliger *et al*., 2015; Harlivasari *et al*., 2024), nicotine dependence (Grant *et al*., 2014), and reduction in cigarettes smoked (Knackstedt *et al*., 2009; Prado *et al*., 2015).

When it comes to alcohol use disorder, a recent trial by Morley et al., has shown that N-acetylcysteine reduced the number of drinks but did not affect craving (Morley *et al*., 2023). Another trial demonstrated that N-acetylcysteine increased the odds of alcohol abstinence, fewer drinks per week, and fewer drinking days per week (Squeglia *et al*., 2018). However, two other studies showed that N-acetylcysteine did not affect drinking outcomes (Stoops *et al*., 2020) (Squeglia *et al*., 2016).

While human clinical results remain inconsistent, two systematic reviews and meta-analyses have concluded the superiority of N-acetylcysteine to placebo in the reduction of cravings among individuals with substance use disorders (Duailibi *et al*., 2017; Chang *et al*., 2021). Although these findings are obtained from the pooled analysis of different abused substances, the compelling outcome stimulates further studies to elucidate the specific and nuanced impact of N-acetylcysteine on craving symptoms in individuals with substance use disorders.

## Mechanistic insights into how N-acetylcysteine can affect drug use relapse and craving

There is evidence on how N-acetylcysteine can potentially contribute to decreasing drug use relapse and craving. Here, we provide potential mechanisms for N-acetylcysteine’s impact on dendritic arborisation, synaptic plasticity, transcriptional downstream targets, and epigenetics, which offer a possible link with ΔFosB-related signalling in drug use relapse and craving (Figure 3).

### N-acetylcysteine rectification of altered dendritic arborizations and synaptic plasticity

The ability of N-acetylcysteine to directly and indirectly modulate glutamatergic neurotransmission, which becomes dysregulated post-withdrawal and contributes to drug-seeking behaviours, *via* interactions with glutamate-transporter type 1 (GLT-1) (Knackstedt *et al*., 2010), as well as NMDARs and AMPARs (Smaga *et al*., 2021b), positions it as a promising candidate for substance use relapse prevention. Reissner et al., reported that in a rat model of cocaine relapse, N-acetylcysteine effectively inhibited cue-induced cocaine reinstatement through the engagement of GLT-1 (Reissner *et al*., 2015). Furthermore, another study also indicated that N-acetylcysteine restores the decrease in GLT-1 levels in both the NAc and dorsal striatum during prolonged but not short cocaine self-administration in rats. This phenomenon was accompanied by an upregulation in the expression of the transcription factor Zif268 (Ducret *et al*., 2016). Moreover, a recent study by Siemsen et al. demonstrated that heroin self-administration and extinction increased prelimbic cortical astrocyte-synapse proximity and circuit-level adaptations in cortical dendritic spine morphology. At the same time, N-acetylcysteine reversed these adaptations in corticostriatal neurons and astrocytes (Siemsen *et al*., 2023).

N-acetylcysteine has also been shown to exhibit a dose-dependent bidirectional effect on the release of dopamine (Gere-Pászti and Jakus, 2009). At the same time, higher doses of N-acetylcysteine hamper striatal dopamine release. Lower doses are associated with an increased release of dopamine in the striatum (Gere-Pászti and Jakus, 2009). Besides, in a primate study, N-acetylcysteine protected against the reduction of striatal dopamine transporter availability, followed by chronic administration of methamphetamine (Hashimoto *et al*., 2004).

The impacts N-acetylcysteine exerts on dopamine availability and the regulation of NMDAR activity can, in part, be germane to facilitating glutathione production (Dean *et al*., 2011). Glutathione can then affect the glutamatergic signalling without altering the level of glutamate (Bradlow *et al*., 2022). Elevated levels of accumbal glutathione in the brains of rats have been reported following treatment with N-acetylcysteine. The increase is believed to result from cell-type specific shifts in glutamatergic inputs to the NAc’s MSNs (Zalachoras *et al*., 2022).

On the other hand, there are studies on the alterations in long-term potentiation (LTP) and long-term depression (LTD) during drug withdrawal or reinstatement. For instance, Qian et al., demonstrated that morphine withdrawal brings about the downregulation of mGluR2/3, impairing NMDA receptor-dependent LTD in the NAc (Qian *et al*., 2019). Moreover, in a study using a reinstatement animal model of drug-seeking behaviour, it was demonstrated that cocaine-induced metaplasticity impaired the induction of LTP and LTD at the PFC-NAc, a phenomenon linked to relapse vulnerability (Moussawi *et al*., 2009). Interestingly, N-acetylcysteine not only prevented relapse and cravings but also restored the induction of LTP. This restoration was presumably achieved through indirect stimulation of mGluRs (mGluR2/3 and mGluR5 primarily), possibly associated with the activation of the cysteine-glutamate exchange system (Moussawi *et al*., 2009). The cysteine-glutamate antiporter regulates glutamate release, which in turn activates presynaptic neuronal mGluR2/3, thereby modulating the vesicular transmission of glutamate (Berk *et al*., 2013).

Taken together, all these lines of evidence suggest that N-acetylcysteine is a potential candidate to rectify the altered glutamatergic and dopaminergic neurotransmission in individuals vulnerable to relapse and reinstatement. The shared dynamics of N-acetylcysteine’s interaction with ΔFosB signalling warrant further research to comprehensively understand its potential therapeutic role.

### N-acetylcysteine impacts on altered transcriptional downstream targets

N-acetylcysteine can be a modulator of redox hemostasis by mitigating the activity of NF-κB through inhibition of IkappaB kinases (Oka *et al*., 2000; Farid *et al*., 2005; Fischer *et al*., 2006; Mackenzie *et al*., 2006). AP-1 has also been demonstrated as a target for N-acetylcysteine, where its activity is diminished via the production of glutathione and the suppression of reactive oxygen species generation (Sen and Packer, 1996; Mackenzie *et al*., 2006; Shi *et al*., 2017). A recent study has also provided evidence that N-acetylcysteine is also capable of diminishing and normalising the hyperactivity of Cdk5, showcasing therapeutic potential in disorders where the excessive enzymatic activity of Cdk5 is implicated (Saha *et al*., 2023), including relapse to substance use and reinstatement behaviour.

Considering the significance of these three downstream entities of ΔFosB in the pathophysiology of relapse, N-acetylcysteine has the potential to reverse the elevated levels of CREB and Cdk5 induced by the overexpression of AP-1 while also addressing heightened levels of NF-κB and Cdk5, which have established roles in craving and drug-seeking behaviour.

### N-acetylcysteine’s impact on epigenetics

Given the role of epigenetics in the pathophysiology of relapse, it is of note to mention that in mammalian cells, histone H3 undergoes S-glutathionylation, introducing glutathione as a novel post-translational participant of the modification of the histone code (García-Giménez *et al*., 2017). However, glutathione’s impact on epigenetic processes goes far beyond H3, and a substantial body of evidence supports the notion that glutathione plays a significant role in epigenetic regulation at various levels (García-Giménez *et al*., 2017). This includes its influence on DNA methylation, histone post-translational modification, non-coding RNAs, and even modulation of chromatin structure (Cyr and Domann, 2011; García-Giménez *et al*., 2017)

This regulatory effect of glutathione on epigenetic processes positions N-acetylcysteine, its precursor, as a prospective candidate for potential epigenetic dysregulation influenced by substance use disorders and implicated in the pathophysiology of relapse and cravings. Nevertheless, it is crucial to emphasise that further in-depth explorative and confirmatory studies are needed to assess N-acetylcysteine’s efficacy and involvement in this context fully. The establishment of a robust scientific foundation through comprehensive research may help determine whether N-acetylcysteine could act as a potential therapeutic tool for modifying altered epigenetic processes induced by substance use disorders.

## Concluding remarks

Relapse and reinstatement to drugs of abuse, even after successfully quitting them, is a deadlock to reaching the ultimately desired treatment goals in substance use disorders. To overcome the relapse, the biological components behind such an unbridled craving should intensively be sought. ΔFosB is one of the presumed candidates responsible for morphological and psychobiological alterations in an addicted brain. In specific brain parts, ΔFosB can modulate several downstream transcriptional factors, such as NF-κB and Cdk5. It also affects AMPA and NMDA receptors through glutamatergic neurotransmission and controls synaptic plasticity. One other explanation of how ΔFosB leaves such permanent effects is epigenetic modifications. Though we have good evidence to rely on, there is much ground to be made up, and further studies are needed to illuminate the role of ΔFosB in relapse and craving comprehensively (Question Box).

**Table:** 

Question box
Can keeping the NAc’s CREB level high, and preventing the CREB’s level from declining in the NAc be a potential target to reduce the rate of relapse after reinstatement (by affecting dynorphin activity)? Is ΔFosB’s effects on relapse to substance use sex-specific? What can be the controlling mechanisms? How can the induction of ΔFosB in different subtypes of MSNs influence its mode of action? Can having the phosphorylation of ΔFosB targeted therapeutically reduce the relapse by reducing the half-life of ΔFosB? Can alteration of ΔFosB’s dimerization pattern be of potential therapeutic importance? How can CaMKII be selectively modulated in substance use disorders? Can modulation of ΔFosB’s production through upstream signalling (CREB, SRF, ALK4, PCBP1, etc) be of therapeutic value in relapse to drugs of abuse? How significant is the role of NMDARs in relapse and can alteration of long-term potentiation be targeted to reduce the craving and reinstatememnt? Is there a possible correlation between diminished MEF2 and increased expression of ΔFosB in substance use disorders? Can epigenetic alteration of chromatin accessibility be a promising therapeutic target in substance use disorders? Do different levels of steroid hormones affect the liability to relapse in substance use disorders by changing chromatin accessibility? Is ΔFosB’s distinct effect in sexes controlled with sex hormones effect on chromatin remodeling? What are the roles of silent synapses in the pathophysiology of relapse and how does ΔFosB modulate them?

Knowing the intricate roles and mechanisms behind ΔFosB’s mode of action in substance use disorders, we might be able to target several upstream and downstream related components to obliterate the associated memory with drugs of abuse and introduce a new treatment strategy to avoid relapse and reinforcement. N-acetylcysteine has demonstrated effectiveness in preventing relapse, reducing craving, and curtailing drug-seeking behaviours in both preclinical and clinical studies, although some discrepancies exist. Due to its potential to modulate glutamatergic and dopaminergic neurotransmission, along with its ability to affect downstream signalling transcription factors and pathways altered by ΔFosB, N-acetylcysteine holds promise as a potential treatment strategy for preventing relapse to substance use. Nevertheless, a more extensive body of both preclinical and clinical research is warranted to establish its efficacy and unravel the elusive mechanisms underlying its effects. Its well-tolerated nature, safety profile, and accessibility make it an ideal candidate for patients with substance use disorder who are already at risk of toxicity and overdose.

## References

[ref1] Alibhai IN , Green TA , Potashkin JA and Nestler EJ (2007) Regulation of fosB and ΔfosB mRNA expression: in vivo and in vitro studies. Brain Research 1143, 22–33. DOI: 10.1016/j.brainres.2007.01.069. 17324382 PMC1880876

[ref2] Amen SL , Piacentine LB , Ahmad ME , Li S-J , Mantsch JR , Risinger RC and Baker DA (2011) Repeated N-acetyl cysteine reduces cocaine seeking in rodents and craving in cocaine-dependent humans. Neuropsychopharmacology 36, 871–878. 21160464 10.1038/npp.2010.226 PMC3052624

[ref3] Ang E , Chen J , Zagouras P , Magna H , Holland J , Schaeffer E and Nestler EJ (2008) Induction of nuclear factor-κB in nucleus accumbens by chronic cocaine administration. Journal of Neurochemistry 79, 221–224. DOI: 10.1046/j.1471-4159.2001.00563.x. 11595774

[ref4] Arancini L , Mohebbi M , Berk M , Dean OM , Bortolasci CC , Spolding B , Zazula R and Dodd S (2021) A placebo-controlled, randomised pilot trial of N-acetylcysteine or placebo for cessation of tobacco smoking. European Neuropsychopharmacology 53, 120–126. 34757312 10.1016/j.euroneuro.2021.10.002

[ref5] Back SE , McCauley JL , Korte KJ , Gros DF , Leavitt V , Gray KM , Hamner MB , DeSantis SM , Malcolm R and Brady KT (2016) A double-blind, randomized, controlled pilot trial of N-acetylcysteine in veterans with posttraumatic stress disorder and substance use disorders. J Clin Psychiatry 77, 1–2406. 27736051 10.4088/JCP.15m10239 PMC5226873

[ref6] Bali P and Kenny PJ (2019) Transcriptional mechanisms of drug addiction. Dialogues in Clinical Neuroscience 21(4), 379–387. 31949405 10.31887/DCNS.2019.21.4/pkenny PMC6952748

[ref7] Becker JB and Koob GF (2016) Sex differences in animal models: focus on addiction. Pharmacological Reviews 68, 242–263. 26772794 10.1124/pr.115.011163 PMC4813426

[ref8] Benavides DR and Bibb JA (2004) Role of Cdk5 in drug abuse and plasticity. Annals of the New York Academy of Sciences, 1025(1), 335–344. DOI: 10.1196/annals.1316.041. 15542734

[ref9] Berk M , Malhi GS , Gray LJ and Dean OM (2013) The promise of N-acetylcysteine in neuropsychiatry. Trends in Pharmacological Sciences 34, 167–177. 23369637 10.1016/j.tips.2013.01.001

[ref10] Bibb JA , Chen J , Taylor JR , Svenningsson P , Nishi A , Snyder GL , Yan Z , Sagawa ZK , Ouimet CC , Nairn AC , Nestler EJ and Greengard P (2001) Effects of chronic exposure to cocaine are regulated by the neuronal protein Cdk5. Nature 410, 376–380. DOI: 10.1038/35066591. 11268215

[ref11] Boersma MCH , Dresselhaus EC , de Biase LM , Mihalas AB , Bergles DE and Meffert MK (2011) A requirement for nuclear factor-κB in developmental and plasticity-associated synaptogenesis. Journal of Neuroscience 31, 5414–5425. DOI: 10.1523/JNEUROSCI.2456-10.2011. 21471377 PMC3113725

[ref12] Bradlow RCJ , Berk M , Kalivas PW , Back SE and Kanaan RA (2022) The potential of N-acetyl-L-cysteine (NAC) in the treatment of psychiatric disorders. CNS Drugs 36, 451–482. 35316513 10.1007/s40263-022-00907-3 PMC9095537

[ref13] Bryant CD and Yazdani N (2016) RNA-binding proteins, neural development and the addictions. Genes Brain and Behavior 15, 169–186. DOI: 10.1111/gbb.12273. 26643147 PMC4944654

[ref14] Cadet JL (2021) Sex in the nucleus accumbens: ΔFosB, addiction, and affective states. Biological Psychiatry 90, 508–510. 34556203 10.1016/j.biopsych.2021.08.002

[ref15] Cano-Cebrián MJ , Fernández-Rodríguez S , Hipólito L , Granero L , Polache A and Zornoza T (2021) Efficacy of N-acetylcysteine in the prevention of alcohol relapse-like drinking: study in long-term ethanol-experienced male rats. Journal of Neuroscience Research 99, 638–648. 33063355 10.1002/jnr.24736

[ref16] Carle TL , Ohnishi YN , Ohnishi YH , Alibhai IN , Wilkinson MB , Kumar A and Nestler EJ (2007) Proteasome-dependent and -independent mechanisms for fosB destabilization: identification of fosB degron domains and implications for ΔFosB stability. European Journal of Neuroscience 25, 3009–3019. DOI: 10.1111/j.1460-9568.2007.05575.x. 17561814

[ref17] Carneiro De Oliveira PE , Leão RM , Bianchi PC , Marin MT , Da Silva Planeta C and Cruz FC (2016) Stress-induced locomotor sensitization to amphetamine in adult, but not in adolescent rats, is associated with increased expression of ΔFosB in the nucleus accumbens. Frontiers in Behavioral Neuroscience 10, 173. DOI: 10.3389/FNBEH.2016.00173/BIBTEX. 27672362 PMC5018519

[ref18] Chang C-T , Hsieh P-J , Lee H-C , Lo C-H , Tam K-W and Loh E-W (2021) Effectiveness of N-acetylcysteine in treating clinical symptoms of substance abuse and dependence: a meta-analysis of randomized controlled trials. Clinical Psychopharmacology and Neuroscience 19, 282–293. 33888657 10.9758/cpn.2021.19.2.282 PMC8077050

[ref19] Chapp AD , Nwakama CA , Jagtap PP , Phan C-MH , Thomas MJ and Mermelstein PG (2024) Fundamental sex differences in cocaine-induced plasticity of dopamine D1 receptor-and D2 receptor-expressing medium spiny neurons in the mouse nucleus accumbens shell. Biological Psychiatry Global Open Science 4, 100295. 38533248 10.1016/j.bpsgos.2024.100295 PMC10963205

[ref20] Chen J , Zhang Y , Kelz MB , Steffen C , Ang ES , Zeng L and Nestler EJ (2000) Induction of cyclin-dependent kinase 5 in the hippocampus by chronic electroconvulsive seizures: role of ΔFosB. Journal of Neuroscience 20, 8965–8971. DOI: 10.1523/jneurosci.20-24-08965.2000. 11124971 PMC6773018

[ref21] Colby CR , Whisler K , Steffen C , Nestler EJ and Self DW (2003) Striatal cell type-specific overexpression of ΔFosB enhances incentive for cocaine. Journal of Neuroscience 23, 2488–2493. DOI: 10.1523/jneurosci.23-06-02488.2003. 12657709 PMC6742034

[ref22] Conrad KL , Tseng KY , Uejima JL , Reimers JM , Heng L-J , Shaham Y , Marinelli M and Wolf ME (2008) Formation of accumbens GluR2-lacking AMPA receptors mediates incubation of cocaine craving. Nature 454, 118–121. 18500330 10.1038/nature06995 PMC2574981

[ref23] Cyr AR and Domann FE (2011) The redox basis of epigenetic modifications: from mechanisms to functional consequences. Antioxid Redox Signal 15, 551–589. 20919933 10.1089/ars.2010.3492 PMC3118659

[ref24] Daiwile AP , Jayanthi S and Cadet JL (2022a) Sex differences in methamphetamine use disorder perused from pre-clinical and clinical studies: potential therapeutic impacts. Neuroscience & Biobehavioral Reviews 137, 104674. 35452744 10.1016/j.neubiorev.2022.104674 PMC9119944

[ref25] Daiwile AP , Sullivan P , Jayanthi S , Goldstein DS and Cadet JL (2022b) Sex-specific alterations in dopamine metabolism in the brain after methamphetamine self-administration. International Journal of Molecular Sciences 23, 4353. 35457170 10.3390/ijms23084353 PMC9027322

[ref26] Daneff M and Jadavji N (2019) The role of synaptic plasticity in the pathophysiology of cocaine addiction. Journal of Young Investigators 37, 33–38. DOI: 10.22186/jyi.37.4.33-38.

[ref27] Dean O , Giorlando F and Berk M (2011) N-acetylcysteine in psychiatry: current therapeutic evidence and potential mechanisms of action. Journal of Psychiatry and Neuroscience 36, 78–86. 21118657 10.1503/jpn.100057 PMC3044191

[ref28] Dresselhaus EC , Boersma MCH and Meffert MK (2018) Targeting of NF-κB to dendritic spines is required for synaptic signaling and spine development. Journal of Neuroscience 38, 4093–4103. DOI: 10.1523/JNEUROSCI.2663-16.2018. 29555853 PMC5963848

[ref29] Duailibi MS , Cordeiro Q , Brietzke E , Ribeiro M , LaRowe S , Berk M and Trevizol AP (2017) N-acetylcysteine in the treatment of craving in substance use disorders: systematic review and meta-analysis. The American Journal on Addictions 26, 660–666. 28898494 10.1111/ajad.12620

[ref30] Ducret E , Puaud M , Lacoste J , Belin-Rauscent A , Fouyssac M , Dugast E , Murray JE , Everitt BJ , Houeto J-L and Belin D (2016) N-acetylcysteine facilitates self-imposed abstinence after escalation of cocaine intake. Biological Psychiatry 80, 226–234. 26592462 10.1016/j.biopsych.2015.09.019 PMC4954758

[ref31] Eagle A , Al Masraf B and Robison AJ (2019) Transcriptional and epigenetic regulation of reward circuitry in drug addiction. In Mary T (ed), Neural Mechanisms of Addiction. Cambridge: Academic Press, pp. 23–34. DOI: 10.1016/b978-0-12-812202-0.00003-8.

[ref32] Farid M , Reid MB , Li Y-P , Gerken E and Durham WJ (2005) Effects of dietary curcumin or N-acetylcysteine on NF-κB activity and contractile performance in ambulatory and unloaded murine soleus. Nutrition & Metabolism 2, 1–8. 16124875 10.1186/1743-7075-2-20 PMC1208951

[ref33] Fischer UM , Antonyan A , Bloch W and Mehlhorn U (2006) Impact of antioxidative treatment on nuclear factor kappa-B regulation during myocardial ischemia-reperfusion. Interactive CardioVascular and Thoracic Surgery 5, 531–535. 17670639 10.1510/icvts.2006.130765

[ref34] Fredriksson I , Jayaram-Lindström N , Kalivas PW , Melas PA and Steensland P (2023) N-acetylcysteine improves impulse control and attenuates relapse-like alcohol intake in long-term drinking rats. Behavioural Brain Research 436, 114089. 36063970 10.1016/j.bbr.2022.114089

[ref35] Froeliger B , McConnell PA , Stankeviciute N , McClure EA , Kalivas PW and Gray KM (2015) The effects of N-acetylcysteine on frontostriatal resting-state functional connectivity, withdrawal symptoms and smoking abstinence: a double-blind, placebo-controlled fMRI pilot study. Drug and Alcohol Dependence 156, 234–242. 26454838 10.1016/j.drugalcdep.2015.09.021 PMC4633320

[ref36] Gajewski PA , Turecki G and Robison AJ (2016) Differential expression of FosB proteins and potential target genes in select brain regions of addiction and depression patients. PLoS One 11, e0160355. DOI: 10.1371/journal.pone. 27494187 PMC4975388

[ref37] García-Giménez JL , Roma-Mateo C , Perez-Machado G , Peiro-Chova L and Pallardó FV (2017) Role of glutathione in the regulation of epigenetic mechanisms in disease. Free Radical Biology and Medicine 112, 36–48. 28705657 10.1016/j.freeradbiomed.2017.07.008

[ref38] Garcia-Keller C , Smiley C , Monforton C , Melton S , Kalivas PW and Gass J (2020) N-acetylcysteine treatment during acute stress prevents stress-induced augmentation of addictive drug use and relapse. Addiction Biology 25, e12798. 31282090 10.1111/adb.12798 PMC7439767

[ref39] Gere-Pászti E and Jakus J (2009) The effect of N-acetylcysteine on amphetamine-mediated dopamine release in rat brain striatal slices by ion-pair reversed-phase high performance liquid chromatography. Biomedical Chromatography 23, 658–664. 19277967 10.1002/bmc.1171

[ref40] Goeders NE (2003) The impact of stress on addiction. European Neuropsychopharmacology 13, 435–441. DOI: 10.1016/J.EURONEURO.2003.08.004. 14636959

[ref41] Goodman J and Packard MG (2016) Memory systems and the addicted brain. Frontiers in Psychiatry 7, 24. DOI: 10.3389/fpsyt.2016.00024. 26941660 PMC4766276

[ref42] Grant JE , Odlaug BL , Chamberlain SR , Potenza MN , Schreiber LRN , Donahue CB and Kim SW (2014) A randomized, placebo-controlled trial of N-acetylcysteine plus imaginal desensitization for nicotine-dependent pathological gamblers. The Journal of Clinical Psychiatry 75, 39–45. DOI: 10.4088/JCP.13M08411. 24345329

[ref43] Gray KM , Carpenter MJ , Baker NL , DeSantis SM , Kryway E , Hartwell KJ , McRae-Clark AL and Brady KT (2012) A double-blind randomized controlled trial of N-acetylcysteine in cannabis-dependent adolescents. American Journal of Psychiatry 169, 805–812. DOI: 10.1176/APPI.AJP.2012.12010055/ASSET/IMAGES/LARGE/AJP.169.8.805.F003.JPEG. 22706327 PMC3410961

[ref44] Gray KM , Sonne SC , McClure EA , Ghitza UE , Matthews AG , McRae-Clark AL , Carroll KM , Potter JS , Wiest K , Mooney LJ , Hasson A , Walsh SL , Lofwall MR , Babalonis S , Lindblad RW , Sparenborg S , Wahle A and King JS (2017) A randomized placebo-controlled trial of N-acetylcysteine for cannabis use disorder in adults. Drug and Alcohol Dependence 177, 249–257. DOI: 10.1016/J.DRUGALCDEP.2017.04.020. 28623823 PMC5535813

[ref45] Grueter BA , Robison AJ , Neve RL , Nestler EJ and Malenka RC (2013) δFosB differentially modulates nucleus accumbens direct and indirect pathway function. Proceedings of the National Academy of Sciences 110, 1923–1928. DOI: 10.1073/pnas.1221742110. PMC3562792 23319622

[ref46] Harlivasari A , Susanto A , Taufik F and Cureus TG- (2024, undefined, 2024) The role of twice-daily N-acetylcysteine (NAC) 2400 mg in smoking cessation: a randomized, placebo-controlled trial in Indonesia, Cureus.comAD harlivasari, AD Susanto, FF Taufik, TT ginting, ADA Susanto sr, FF Taufik, T gintingCureus, 2024 DOI: 10.7759/cureus.54322. PMC10944675 38500894

[ref47] Hashimoto K , Tsukada H , Nishiyama S , Fukumoto D , Kakiuchi T , Shimizu E and Iyo M (2004) Effects of N-acetyl-l-cysteine on the reduction of brain dopamine transporters in monkey treated with methamphetamine. Annals of the New York Academy of Sciences 1025, 231–235. 15542721 10.1196/annals.1316.028

[ref48] Jastrzębska J , Frankowska M , Filip M and Atlas D (2016) N-acetylcysteine amide (AD4) reduces cocaine-induced reinstatement. Psychopharmacology (Berl) 233, 3437–3448. 27469021 10.1007/s00213-016-4388-5

[ref49] Kanaan RA , Oliver G , Dharan A , Sendi S , Maier A , Mohebbi M , Ng C , Back SE , Kalivas P and Berk M (2023) A multi-centre, double-blind, 12-week, randomized, placebo-controlled trial of adjunctive N-acetylcysteine for treatment-resistant PTSD. Psychiatry Research 327, 115398. 37540942 10.1016/j.psychres.2023.115398

[ref50] Kelz MB , Chen J , Carlezon WA , Whisler K , Gilden L , Beckmann AM , Steffen C , Zhang YJ , Marotti L , Self DW , Tkatch T , Baranauskas G , Surmeler DJ , Neve RL , Duman RS , Picciotto MR and Nestler EJ (1999) Expression of the transcription factor ΔFosB in the brain controls sensitivity to cocaine. Nature 401, 272–276. DOI: 10.1038/45790. 10499584

[ref51] Kelz MB and Nestler EJ (2000) ΔFosB: a molecular switch underlying long-term neural plasticity. Current Opinion in Neurology 13, 715–720. DOI: 10.1097/00019052-200012000-00017. 11148675

[ref52] Kennedy PJ , Feng J , Robison AJ , Maze I , Badimon A , Mouzon E , Chaudhury D , Damez-Werno DM , Haggarty SJ , Han MH , Bassel-Duby R , Olson EN and Nestler EJ (2013) Class i HDAC inhibition blocks cocaine-induced plasticity by targeted changes in histone methylation. Nature Neuroscience 16, 434–440. DOI: 10.1038/nn.3354. 23475113 PMC3609040

[ref53] Knackstedt LA , LaRowe S , Mardikian P , Malcolm R , Upadhyaya H , Hedden S , Markou A and Kalivas PW (2009) The role of cystine-glutamate exchange in nicotine dependence in rats and humans. Biological Psychiatry 65, 841–845. DOI: 10.1016/J.BIOPSYCH.2008.10.040. 19103434 PMC2756612

[ref54] Knackstedt LA , Melendez RI and Kalivas PW (2010) Ceftriaxone restores glutamate homeostasis and prevents relapse to cocaine seeking. Biological Psychiatry 67, 81–84. 19717140 10.1016/j.biopsych.2009.07.018 PMC2795043

[ref55] Krapacher FA , Fernández-Suárez D , Andersson A , Carrier-Ruiz A and Ibáñez CF (2022) Convergent dopamine and ALK4 signaling to PCBP1 controls FosB alternative splicing and cocaine behavioral sensitization. The EMBO Journal 41, 10721. 10.15252/embj.2022110721 PMC10545536 35730718

[ref56] Lardner CK , van der Zee Y , Estill MS , Kronman HG , Salery M , Cunningham AM , Godino A , Parise EM , Kim JH and Neve RL (2021) Gene-targeted, CREB-mediated induction of ΔFosB controls distinct downstream transcriptional patterns within D1 and D2 medium spiny neurons. Biological Psychiatry 90, 540–549. 34425966 10.1016/j.biopsych.2021.06.017 PMC8501456

[ref57] LaRowe SD , Kalivas PW , Nicholas JS , Randall PK , Mardikian PN and Malcolm RJ (2013) A double-blind placebo-controlled trial of N-acetylcysteine in the treatment of cocaine dependence. The American Journal on Addictions 22, 443–452. 23952889 10.1111/j.1521-0391.2013.12034.x PMC4348575

[ref58] LaRowe SD , Mardikian P , Malcolm R , Myrick H , Kalivas P , McFarland K , Saladin M , McRae A and Brady K (2006) Safety and tolerability of N-acetylcysteine in cocaine-dependent individuals. American Journal on Addictions 15, 105–110. DOI: 10.1080/10550490500419169. 16449100 PMC1513138

[ref59] Larson EB , Akkentli F , Edwards S , Graham DL , Simmons DL , Alibhai IN , Nestler EJ and Self DW (2010) Striatal regulation of ΔFosB, FosB, and cFos during cocaine self-administration and withdrawal. Journal of Neurochemistry 115, 112–122. DOI: 10.1111/j.1471-4159.2010.06907.x. 20633205 PMC2939959

[ref60] Lebourgeois S , González-Marín MC , Jeanblanc J , Naassila M and Vilpoux C (2018) Effect of N-acetylcysteine on motivation, seeking and relapse to ethanol self-administration. Addiction Biology 23, 643–652. 28557352 10.1111/adb.12521

[ref61] Lee KW , Kim Y , Kim AM , Helmin K , Nairn AC and Greengard P (2006) Cocaine-induced dendritic spine formation in D1 and D2 dopamine receptor-containing medium spiny neurons in nucleus accumbens. Proceedings of the National Academy of Sciences 103, 3399–3404. DOI: 10.1073/pnas.0511244103. PMC1413917 16492766

[ref62] Lobo MK , Zaman S , Damez-Werno DM , Koo JW , Bagot RC , DiNieri JA , Nugent A , Finkel E , Chaudhury D and Chandra R (2013a) ΔFosB induction in striatal medium spiny neuron subtypes in response to chronic pharmacological, emotional, and optogenetic stimuli. Journal of Neuroscience 33, 18381–18395. 24259563 10.1523/JNEUROSCI.1875-13.2013 PMC3834048

[ref63] Lobo MK , Zaman S , Damez-Werno DM , Koo JW , Bagot RC , DiNieri JA , Nugent A , Finkel E , Chaudhury D and Chandra R (2013b) ΔFosB induction in striatal medium spiny neuron subtypes in response to chronic pharmacological, emotional, and optogenetic stimuli. Journal of Neuroscience 33, 18381–18395. 24259563 10.1523/JNEUROSCI.1875-13.2013 PMC3834048

[ref64] Loganathan K and Ho ETW (2021) Value, drug addiction and the brain. Addictive Behaviors 116, 106816. 33453587 10.1016/j.addbeh.2021.106816

[ref65] Lynch WJ , Nicholson KL , Dance ME , Morgan RW and Foley PL (2010) Animal models of substance abuse and addiction: implications for science, animal welfare, and society. Comparative Medicine 60(3), 177–188. 20579432 PMC2890392

[ref66] Mackenzie GG , Zago MP , Erlejman AG , Aimo L , Keen CL and Oteiza PI (2006) α-lipoic acid and N-acetyl cysteine prevent zinc deficiency-induced activation of NF-κB and AP-1 transcription factors in human neuroblastoma IMR-32 cells. Free Radical Research 40, 75–84. 16298762 10.1080/10715760500312305

[ref67] Madayag A , Lobner D , Kau KS , Mantsch JR , Abdulhameed O , Hearing M , Grier MD and Baker DA (2007) Repeated N-acetylcysteine administration alters plasticity-dependent effects of cocaine. The Journal of Neuroscience 27, 13968–13976. DOI: 10.1523/JNEUROSCI.2808-07.2007. 18094234 PMC2996827

[ref68] Maze I , Covingtoni HE , Dietz DM , Laplant Q , Renthal W , Russo SJ , Mechanic M , Mouzon E , Neve RL , Haggarty SJ , Ren Y , Sampath SC , Hurd YL , Greengard P , Tarakhovsky A , Schaefer A and Nestler EJ (2010) Essential role of the histone methyltransferase G9a in cocaine-induced plasticity. Science 327(5962), 213–216. DOI: 10.1126/science.1179438. 20056891 PMC2820240

[ref69] McClung CA and Nestler EJ (2003) Regulation of gene expression and cocaine reward by CREB and ΔFosB. Nature Neuroscience 6, 1208–1215. DOI: 10.1038/nn1143. 14566342

[ref70] McClure EA , Wahlquist AE and Tomko RL (2021) Evaluating N-acetylcysteine for early and end-of-treatment abstinence in adult cigarette smokers. Drug and Alcohol Dependence 225, 108815. 34171822 10.1016/j.drugalcdep.2021.108815 PMC8282766

[ref71] McKetin R , Dean OM , Turner A , Kelly PJ , Quinn B , Lubman DI , Dietze P , Carter G , Higgs P and Sinclair B (2021) N-acetylcysteine (NAC) for methamphetamine dependence: a randomised controlled trial. EClinicalMedicine 38. 10.1016/j.eclinm.2021.101005 PMC8283342 34308314

[ref72] Mews P , Walker DM and Nestler EJ (2018) Epigenetic priming in drug addiction. Cold Spring Harbor Symposia on Quantitative Biology 83, 131–139. DOI: 10.1101/sqb.2018.83.037663. 30936392 PMC6764605

[ref73] Milton AL and Everitt BJ (2012) The persistence of maladaptive memory: addiction, drug memories and anti-relapse treatments. Neuroscience & Biobehavioral Reviews 36, 1119–1139. DOI: 10.1016/j.neubiorev.2012.01.002. 22285426

[ref74] Minarini A , Ferrari S , Galletti M , Giambalvo N , Perrone D , Rioli G and Galeazzi GM (2017) N-acetylcysteine in the treatment of psychiatric disorders: current status and future prospects. Expert Opinion on Drug Metabolism & Toxicology 13, 279–292. 27766914 10.1080/17425255.2017.1251580

[ref75] Morais-Silva G , Alves GC and Marin MT (2016) N-acetylcysteine treatment blocks the development of ethanol-induced behavioural sensitization and related ΔFosB alterations. Neuropharmacology 110, 135–142. 27401790 10.1016/j.neuropharm.2016.07.009

[ref76] Morley KC , Peruch S , Adams C , Towers E , Tremonti C , Watt J , Jamshidi1 N and Haber PS (2023) N acetylcysteine in the treatment of alcohol use disorder: a randomized, double-blind, placebo-controlled trial. Alcohol and Alcoholism 58, 553–560. 37465907 10.1093/alcalc/agad044

[ref77] Mousavi SG , Salehi M , Peykanpour M , Sichani NK and Maracy M (2015) The efficacy of N-acetylcysteine in the treatment of methamphetamine dependence: a double-blind controlled, crossover study. Archives of Iranian Medicine 18, 28–33. 25556383

[ref78] Moussawi K , Pacchioni A , Moran M , Olive MF , Gass JT , Lavin A and Kalivas PW (2009) N-acetylcysteine reverses cocaine-induced metaplasticity. Nature Neuroscience 12, 182–189. 19136971 10.1038/nn.2250 PMC2661026

[ref79] Muschamp JW and Carlezon WA (2013) Roles of nucleus accumbens CREB and dynorphin in dysregulation of motivation. Cold Spring Harbor Perspectives in Medicine 3, a012005. DOI: 10.1101/cshperspect.a012005. 23293139 PMC3552337

[ref80] Nestler EJ (2012) Transcriptional mechanisms of drug addiction. Clinical Psychopharmacology and Neuroscience 10, 136–143. DOI: 10.9758/cpn.2012.10.3.136. 23430970 PMC3569166

[ref81] Nestler EJ (2008) Transcriptional mechanisms of addiction: Role of ΔFosB, Philosophical Transactions of the Royal Society of London. Series B, Biological Sciences 363(1507), 3245–3255. DOI 10.1098/rstb.2008.0067. 18640924 PMC2607320

[ref82] Nestler EJ (2005) Is there a common molecular pathway for addiction ? Nature Neuroscience 8, 1445–1449. DOI: 10.1038/nn1578. 16251986

[ref83] Nestler EJ (2001) Molecular neurobiology of addiction. American Journal on Addictions 10, 201–217. DOI: 10.1080/105504901750532094. 11579619

[ref84] Nestler EJ , Barrot M and Self DW (2001) ΔFosB: A sustained molecular switch for addiction. Proceedings of the National Academy of Sciences 98, 11042–11046. DOI: 10.1073/pnas.191352698 1046. PMC58680 11572966

[ref85] Nikulina EM , Arrillaga-Romany I , Miczek KA and Hammer RP (2008) Long-lasting alteration in mesocorticolimbic structures after repeated social defeat stress in rats: time course of μ-opioid receptor mRNA and FosB/ΔFosB immunoreactivity. European Journal of Neuroscience 27, 2272–2284. DOI: 10.1111/J.1460-9568.2008.06176.X. 18445218 PMC2442756

[ref86] Oka S , Kamata H , Kamata K , Yagisawa H and Hirata H (2000) N-acetylcysteine suppresses TNF-induced NF-κB activation through inhibition of IκB kinases. FEBS Letters 472, 196–202. 10788610 10.1016/s0014-5793(00)01464-2

[ref87] Olsen CM (2011) Natural rewards, neuroplasticity, and non-drug addictions. Neuropharmacology 61, 1109–1122. DOI: 10.1016/j.neuropharm.2011.03.010 21459101 PMC3139704

[ref88] Perrotti LI , Hadeishi Y , Ulery PG , Barrot M , Monteggia L , Duman RS and Nestler EJ (2004) Induction of ΔFosB in reward-related brain structures after chronic stress. Journal of Neuroscience 24, 10594–10602. DOI: 10.1523/JNEUROSCI.2542-04.2004. 15564575 PMC6730117

[ref89] Perrotti LI , Weaver RR , Robison B , Renthal W , Maze I , Yazdani S , Elmore RG , Knapp DJ , Selley DE , Martin BR , Sim-Selley L , Bachtell RK , Self DW and Nestler EJ (2008) Distinct patterns of ΔFosB induction in brain by drugs of abuse. Synapse 62, 358–369. DOI: 10.1002/syn.20500. 18293355 PMC2667282

[ref90] Prado E , Maes M , Piccoli LG , Baracat M , Barbosa DS , Franco O , Dodd S , Berk M and Nunes SOV (2015) N-acetylcysteine for therapy-resistant tobacco use disorder: a pilot study. Redox Report 20, 215–222. DOI: 10.1179/1351000215Y.0000000004. 25729878 PMC6837411

[ref91] Pulipparacharuvil S , Renthal W , Hale CF , Taniguchi M , Xiao G , Kumar A , Russo SJ , Sikder D , Dewey CM , Davis MM , Greengard P , Nairn AC , Nestler EJ and Cowan CW (2008) Cocaine regulates MEF2 to control synaptic and behavioral plasticity. Neuron 59, 621–633. DOI: 10.1016/j.neuron.2008.06.020. 18760698 PMC2626175

[ref92] Qian Z , Wu X , Qiao Y , Shi M , Liu Z , Ren W , Han J and Zheng Q (2019) Downregulation of mGluR2/3 receptors during morphine withdrawal in rats impairs mGluR2/3-and NMDA receptor-dependent long-term depression in the nucleus accumbens. Neuroscience Letters 690, 76–82. 30315852 10.1016/j.neulet.2018.10.018

[ref93] Reissner KJ , Gipson CD , Tran PK , Knackstedt LA , Scofield MD and Kalivas PW (2015) Glutamate transporter GLT-1 mediates N-acetylcysteine inhibition of cocaine reinstatement. Addiction Biology 20, 316–323. 24612076 10.1111/adb.12127 PMC4437505

[ref94] Robinson TE and Kolb B (1997) Persistent structural modifications in nucleus accumbens and prefrontal cortex neurons produced by previous experience with amphetamine. Journal of Neuroscience 17, 8491–8497. DOI: 10.1523/jneurosci.17-21-08491.1997. 9334421 PMC6573726

[ref95] Robison AJ , Vialou V , Mazei-Robison M , Feng J , Kourrich S , Collins M , Wee S , Koob G , Turecki G , Neve R , Thomas M and Nestler EJ (2013) Behavioral and structural responses to chronic cocaine require a feedforward loop involving Δ FosB and calcium/ calmodulin-dependent protein kinase II in the nucleus accumbens shell. Journal of Neuroscience 33, 4295–4307. DOI: 10.1523/JNEUROSCI.5192-12.2013. 23467346 PMC3658178

[ref96] Saberian H , Taei AA , Torkaman-Boutorabi A , Riahi E , Aminyavari S , Naghizadeh A and Farahmandfar M (2021) Effect of histone acetylation on maintenance and reinstatement of morphine-induced conditioned place preference and ΔFosB expression in the nucleus accumbens and prefrontal cortex of male rats. Behavioural Brain Research 414, 113477. 34302880 10.1016/j.bbr.2021.113477

[ref97] Saha D , Paul S , Gaharwar U , Priya A , Neog A , Singh A and Bk B (2023) Cdk5-mediated brain unfolded protein response upregulation associated with cognitive impairments in Type 2 Diabetes and ameliorative action of NAC. ACS Chemical Neuroscience 14, 2761–2774. 37468304 10.1021/acschemneuro.3c00341

[ref98] Salaya-Velazquez NF , López-Muciño LA , Mejía-Chávez S , Sánchez-Aparicio P , Domínguez-Guadarrama AA and Venebra-Muñoz A (2020) Anandamide and sucralose change ΔFosB expression in the reward system. Neuroreport 31, 240–244. DOI: 10.1097/WNR.0000000000001400. 31923023

[ref99] Scalize Hirata RY , dos Santos TB , de Andrade JS , Le Sueur Maluf L , Antunes HKM , Britto LRG , Céspedes IC and de Barros Viana M (2019) Chronic corticosterone increases ΔFOSB and CRFR1 immunoreactivity in brain regions that modulate aversive conditioning. Behavioural Brain Research 356, 107–119. DOI: 10.1016/j.bbr.2018.08.011. 30118773

[ref100] Schmaal L , Berk L , Hulstijn KP , Cousijn J , Wiers RW and van den Brink W (2011) Efficacy of N-acetylcysteine in the treatment of nicotine dependence: a double-blind placebo-controlled pilot study. European Addiction Research 17, 211–216. 21606648 10.1159/000327682

[ref101] Schulte MHJ , Goudriaan AE , Kaag AM , Kooi DP , Van Den Brink W , Wiers RW and Schmaal L (2017) The effect of N-acetylcysteine on brain glutamate and gamma-aminobutyric acid concentrations and on smoking cessation: a randomized, double-blind, placebo-controlled trial. Journal of Psychopharmacology 31, 1377–1379. 28922968 10.1177/0269881117730660 PMC5639948

[ref102] Schulte MHJ , Wiers RW , Boendermaker WJ , Goudriaan AE , van den Brink W , van Deursen DS , Friese M , Brede E and Waters AJ (2018) The effect of N-acetylcysteine and working memory training on cocaine use, craving and inhibition in regular cocaine users: correspondence of lab assessments and ecological momentary assessment. Addictive Behaviors 83, 79–86. 29661657 10.1016/j.addbeh.2018.03.023

[ref103] Seltenhammer MH , Resch U , Stichenwirth M , Seigner J , Reisinger CM , Vycudilik C and W (2016) Accumulation of highly stable ΔFosB-isoforms and its targets inside the reward system of chronic drug abusers - a source of dependence-memory and high relapse rate? Journal of Addiction Research & Therapy 7, 1–8. DOI: 10.4172/2155-6105.1000297.

[ref104] Sen CK and Packer L (1996) Antioxidant and redox regulation of gene transcription. The FASEB journal 10, 709–720. 8635688 10.1096/fasebj.10.7.8635688

[ref105] Shi H , Gu Y , Xie Z , Zhou QI , Mao G , Lin X , Liu K , Liu Y , Zou B and Zhao J (2017) Mechanism of N-acetyl-cysteine inhibition on the cytotoxicity induced by titanium dioxide nanoparticles in JB6 cells transfected with activator protein-1. Experimental and Therapeutic Medicine 13, 3549–3554. 28588678 10.3892/etm.2017.4415 PMC5450798

[ref106] Siemsen BM , Denton AR , Parrila-Carrero J , Hooker KN , Carpenter EA , Prescot ME , Brock AG , Westphal AM , Leath M-N and McFaddin JA (2023) Heroin self-administration and extinction increase prelimbic cortical astrocyte-synapse proximity and alter dendritic spine morphometrics that are reversed by N-acetylcysteine. Cells 12, 1812. 37508477 10.3390/cells12141812 PMC10378353

[ref107] Silva RJM , Galinato MH and Mandyam C (2016) Role of hippocampal neurogenesis in drug addiction. The FASEB Journal 30, 518 4–518 4. DOI: 10.1096/fasebj.30.1_supplement.518.4.

[ref108] Sinha R (2012) How does stress lead to risk of alcohol relapse? Alcohol Research: Current Reviews 34, 432–440. 23584109 10.35946/arcr.v34.4.07 PMC3788822

[ref109] Sinha R (2008) Chronic stress, drug use, and vulnerability to addiction. Annals of the New York Academy of Sciences 1141, 105–130. DOI: 10.1196/ANNALS.1441.030. 18991954 PMC2732004

[ref110] Smaga I , Frankowska M and Filip M (2021a) N-acetylcysteine in substance use disorder: a lesson from preclinical and clinical research. Pharmacological Reports 73, 1205–1219. 34091880 10.1007/s43440-021-00283-7 PMC8460563

[ref111] Smaga I , Frankowska M and Filip M (2021b) N-acetylcysteine as a new prominent approach for treating psychiatric disorders. British Journal of Pharmacology 178, 2569–2594. 33760228 10.1111/bph.15456

[ref112] Spiga S , Mulas G , Piras F and Diana M (2014) The “addicted” spine. Frontiers in Neuroanatomy 8(OCT), 112491. DOI: 10.3389/fnana.2014.00110. PMC4183114 25324733

[ref113] Squeglia LM , Baker NL , McClure EA , Tomko RL , Adisetiyo V and Gray KM (2016) Alcohol use during a trial of N-acetylcysteine for adolescent marijuana cessation. Addictive Behaviors 63, 172–177. 27521979 10.1016/j.addbeh.2016.08.001 PMC4993655

[ref114] Squeglia LM , Tomko RL , Baker NL , McClure EA , Book GA and Gray KM (2018) The effect of N-acetylcysteine on alcohol use during a cannabis cessation trial. Drug and Alcohol Dependence 185, 17–22. 29413434 10.1016/j.drugalcdep.2017.12.005 PMC5889716

[ref115] Stewart AF , Fulton SL and Maze I (2021) Epigenetics of drug addiction. Cold Spring Harbor Perspectives in Medicine 11(7), a040253. DOI: 10.1101/CSHPERSPECT.A040253. 32513670 PMC7967246

[ref116] Stoops WW , Strickland JC , Hays LR , Rayapati AO , Lile JA and Rush CR (2020) Influence of n-acetylcysteine maintenance on the pharmacodynamic effects of oral ethanol. Pharmacology Biochemistry and Behavior 198, 173037. 32891709 10.1016/j.pbb.2020.173037 PMC7471929

[ref117] Takahashi M , Nakabayashi T , Mita N , Jin X , Aikawa Y , Sasamoto K , Miyoshi G , Miyata M , Inoue T and Ohshima T (2022) Involvement of Cdk5 activating subunit p35 in synaptic plasticity in excitatory and inhibitory neurons. Molecular Brain 15, 37. DOI: 10.1186/s13041-022-00922-x. 35484559 PMC9052517

[ref118] Teague CD and Nestler EJ (2021) Key transcription factors mediating cocaine-induced plasticity in the nucleus accumbens. Molecular Psychiatry 1–23, 34079067 10.1038/s41380-021-01163-5 PMC8636523

[ref119] Todtenkopf MS , Parsegian A , Naydenov A , Neve RL , Konradi C and Carlezon WA (2006) Brain reward regulated by AMPA receptor subunits in nucleus accumbens shell. Journal of Neuroscience 26, 11665–11661. DOI: 10.1523/JNEUROSCI.3070-06.2006. 17093088 PMC4205583

[ref120] Torres OV , Mccoy MT , Ladenheim B , Jayanthi S , Brannock C , Tulloch I , Krasnova IN and Cadet JL (2015) CAMKII-conditional deletion of histone deacetylase 2 potentiates acute methamphetamine-induced expression of immediate early genes in the mouse nucleus accumbens. Scientific Reports 5, 1–11. DOI: 10.1038/srep13396. PMC4547138 26300473

[ref121] Ulery PG , Rudenko G and Nestler EJ (2006) Regulation of ΔFosB stability by phosphorylation. Journal of Neuroscience 26, 5131–5142. DOI: 10.1523/JNEUROSCI.4970-05.2006. 16687504 PMC6674239

[ref122] Vialou V , Feng J , Robison AJ , Ku SM , Ferguson D , Scobie KN , Mazei-Robison MS , Mouzon E and Nestler EJ (2012) Serum response factor and cAMP response element binding protein are both required for cocaine induction of ΔFosB. Journal of Neuroscience 32, 7577–7584. DOI: 10.1523/JNEUROSCI.1381-12.2012. 22649236 PMC3370956

[ref123] Vialou V , Robison AJ , Laplant QC , Covington HE , Dietz DM , Ohnishi YN , Mouzon E , Rush AJ , Watts EL , Wallace DL , Ĩiguez SD , Ohnishi YH , Steiner MA , Warren BL , Krishnan V , Bolãos CA , Neve RL , Ghose S , Berton O , Tamminga CA and Nestler EJ (2010) ΔfosB in brain reward circuits mediates resilience to stress and antidepressant responses. Nature Neuroscience 13, 745–752. DOI: 10.1038/nn.2551. 20473292 PMC2895556

[ref124] Volkow ND and Morales M (2015) The brain on drugs: from reward to addiction. Cell 162, 712–725. DOI: 10.1016/j.cell.2015.07.046 26276628

[ref125] Wallace DL , Vialou V , Rios L , Carle-Florence TL , Chakravarty S , Kumar A , Graham DL , Green TA , Kirk A , Iñiguez SD , Perrotti LI , Barrot M , DiLeone RJ , Nestler EJ and Bolaños-Guzmán CA (2008) The influence of ΔfosB in the nucleus accumbens on natural reward-related behavior. Journal of Neuroscience 28, 10272–10277. DOI: 10.1523/JNEUROSCI.1531-08.2008. 18842886 PMC2653197

[ref126] Wang Y , Cesena TI , Ohnishi Y , Burger-Caplan R , Lam V , Kirchhoff PD , Larsen SD , Larsen MJ , Nestler EJ and Rudenko G (2012) Small molecule screening identifies regulators of the transcription factor ΔfosB. ACS Chemical Neuroscience 3, 546–556. DOI: 10.1021/cn3000235. 22860224 PMC3399579

[ref127] Wassum KM and Izquierdo A (2015) The basolateral amygdala in reward learning and addiction. Neuroscience & Biobehavioral Reviews 57, 271–283. DOI: 10.1016/j.neubiorev.2015.08.017. 26341938 PMC4681295

[ref128] Wen D , Hui R , Liu Y , Luo Y , Wang J , Shen X , Xie B , Yu F , Cong B and Ma C (2020) Molecular hydrogen attenuates methamphetamine-induced behavioral sensitization and activation of ERK-ΔFosB signaling in the mouse nucleus accumbens. Progress in Neuro-Psychopharmacology & Biological Psychiatry 97, 109781. DOI: 10.1016/j.pnpbp.2019.109781. 31629777

[ref129] Willuhn I , Wanat MJ , Clark JJ and Phillips PEM (2010) Dopamine signaling in the nucleus accumbens of animals self-administering drugs of abuse. Current Topics in Behavioral Neurosciences 3, 29–71. DOI: 10.1007/7854_2009_27 21161749 PMC3766749

[ref130] Wimmer ME , Fant B , Swinford-Jackson SE , Testino A , Van Nest D , Abel T and Pierce RC (2019) H3. 3 barcoding of nucleus accumbens transcriptional activity identifies novel molecular cascades associated with cocaine self-administration in mice. Journal of Neuroscience 39, 5247–5254. 31043484 10.1523/JNEUROSCI.0015-19.2019 PMC6607753

[ref131] Woodcock EA , Lundahl LH , Khatib D , Stanley JA and Greenwald MK (2021) N-acetylcysteine reduces cocaine-seeking behavior and anterior cingulate glutamate/glutamine levels among cocaine-dependent individuals. Addiction Biology 26, e12900. DOI: 10.1111/ADB. 32212237 PMC10369173

[ref132] Yeh S-Y , Estill M , Lardner CK , Browne CJ , Minier-Toribio A , Futamura R , Beach K , McManus CA , Xu S and Zhang S (2023) Cell type-specific whole-genome landscape of ΔFOSB binding in the nucleus accumbens after chronic cocaine exposure. Biological Psychiatry 94, 367–377. 36906500 10.1016/j.biopsych.2022.12.021 PMC10314970

[ref133] Yin Z , Venkannagari H , Lynch H , Aglyamova G , Bhandari M , Machius M , Nestler EJ , Robison AJ and Rudenko G (2020) Self-assembly of the bZIP transcription factor ΔFosB. Current Research in Structural Biology 2, 1–13. 32542236 10.1016/j.crstbi.2019.12.001 PMC7295165

[ref134] Zachariou V , Bolanos CA , Selley DE , Theobald D , Cassidy MP , Kelz MB , Shaw-Lutchman T , Berton O , Sim-Selley LJ , Dileone RJ , Kumar A and Nestler EJ (2006) An essential role for ΔFosB in the nucleus accumbens in morphine action. Nature Neuroscience 9, 205–211. DOI: 10.1038/nn1636. 16415864

[ref135] Zalachoras I , Ramos-Fernández E , Hollis F , Trovo L , Rodrigues J , Strasser A , Zanoletti O , Steiner P , Preitner N and Xin L (2022) Glutathione in the nucleus accumbens regulates motivation to exert reward-incentivized effort. Elife 11, e77791. 36345724 10.7554/eLife.77791 PMC9642999

[ref136] Zhang Lei , Huang L , Lu K , Liu Y , Tu G , Zhu M , Ying L , Zhao J , Liu N , Guo F , Zhang Lin and Zhang Lu (2017) Cocaine-induced synaptic structural modification is differentially regulated by dopamine D1 and D3 receptors-mediated signaling pathways. Addiction Biology 22, 1842–1855. DOI: 10.1111/adb.12462 27734601

[ref137] Zhang Y , Crofton EJ , Li D , Lobo MK , Fan X , Nestler EJ and Green TA (2014) Overexpression of deltaFosB in nucleus accumbens mimics the protective addiction phenotype, but not the protective depression phenotype of environmental enrichment. Frontiers in Behavioral Neuroscience 8, 297. DOI: 10.3389/fnbeh.2014.00297. 25221490 PMC4148937

[ref138] Zhang Z and Zhao Y (2022) Progress on the roles of MEF2C in neuropsychiatric diseases. Molecular Brain 15, 1–11. 34991657 10.1186/s13041-021-00892-6 PMC8740500

